# Reproduction system development of *Ceracris kiangsu* Tsai female adults and its relationship with fitness characteristics

**DOI:** 10.3389/fphys.2023.1136559

**Published:** 2023-03-07

**Authors:** Meizhi Wang, Hongmei Li, Wei Zhang, Fuyan Zhuo, Tianjiao Li, Alyssa Lowry, Aihuan Zhang

**Affiliations:** ^1^ College of Bioscience and Resource Environment, Beijing University of Agriculture, Beijing, China; ^2^ MARA-CABI Joint Laboratory for Bio-safety, Institute of Plant Protection, Chinese Academy of Agricultural Science, Beijing, China; ^3^ CABI East and Southeast Asia, Beijing, China; ^4^ Research Institute of Subtropical Forestry/Chinese Academy of Forestry, Fuyang, China; ^5^ National Agro-Tech Extension and Service Center, Beijing, China; ^6^ CAB International, Egham, United Kingdom

**Keywords:** yellow-spined bamboo locust, phenotypic traits, ovary, grade, migratory pest, dissection

## Abstract

Research on the ovarian development of insect pests helps provide key information for predicting pest occurrences, and currently, there is very limited information about the reproductive system of *Ceracris kiangsu* Tsai. This study aimed to assess the reproductive fitness of 321 adult female insects by using traditional methods to dissect female adults, measure female ovaries, and assess the process of egg formation. The phenotypic traits including body weight and body length were also measured and used to estimate the model of ovarian developmental stages. Four ovarian developmental stages before the oviposition were identified, and the fundamental ovarian structure of *C. kiangsu* displayed red dots on the matured eggs inside the calyx at ovarian developmental stage V. The accessory glands of *C. kiangsu* had the deepest folds at stage Ⅲ. Redundancy analysis (RDA) was used to explore the correlation between ovarian development, body weight, and body length. A significant positive correlation was observed for body weight (*p* = 0.001) and body length (*p* = 0.009), which varied with the grade of ovarian development evaluated by the ovarian developmental stage, ovarian length, ovarian width, and ovarian cross-sectional area. A partial least square (PLS) regression was used to model the ovarian developmental stage, with a stage-based PLS being identified as the more effective method, which was *y* = 1.509*x*_1_ + 0.114*x*_2_. The model provides a potentially rapid way to identify the population source as either “native” or “immigrant” from the phenotypic traits without dissection. The aforementioned model may be used to estimate adult emergence periods and identify migratory populations from their ovarian development, potentially aiding in implementing proper prevention measures.

## 1 Introduction

The monitoring and prediction of insect pests is fundamental to conducting effective pest management. Current measures and approaches for the prediction of pest occurrences include, but is not limited to, light traps, sex pheromone traps, and field monitoring. Another technique that has been used to predict pest population dynamics and occurrences in migrating species is the dissection of adult female insects (Qi et al., 2011). This technique has been shown to provide information on not only the ovarian development but also the peak oviposition period, which can then be used to estimate the reproductive capacity of the population and the migration potential of the pest (Liu and Huang, 2018). Research studies have been conducted on ovarian structures (Hodge, 1943; Liu and Lu, 1959; Snodgrass, 1993), ovarian developmental stages (Wu and Zhang, 1997; Chen et al., 2005; Ren et al., 2014; Zhang et al., 2020), and the effects of insect hormones and miRNAs on ovarian development. A good example of this is the juvenile hormones of *Locusta migratoria manilensis* (Meyen) in which Let-7 and miRNA-278 have been shown to play a vital role in regulating its reproductive system (Song et al., 2013; Song et al., 2019). However, external factors have also been shown to affect ovarian development; Bhalerao et al. (1987) found that the feeding reduction had a negative effect on the vitellogenesis of *Euscelimena harpago* (Serville) and *Potua sabulosa* Hancock female locusts’ ovaries. Chen et al. (2008) found that the host plants had a positive effect on ovarian development and fecundity of *Fruhstorferiola tonkinensis* Willemse. Additionally, mating has also been shown to exert a positive effect on the developmental phenotype of *Eriopis connexa* (Germar) offspring and on the increase in the proportion of mature eggs in female *Aedes taeniorhynchus* (Wiedemann). Mating was also found to accelerate both ovarian development and reproduction abilities of female *Apolygus lucorum* (Meyer-Dür) (Meara and Evans, 1977; Colares et al., 2015; Li et al., 2017). Whilst the extreme temperature and short photoperiod reduced the fecundity of *Drosophila suzukii* (Matsumura) and *Athetis lepigone* Möschler (Everman et al., 2018; Liang et al., 2021), the ovarian development of *Spodoptera mauritia* (Boisduval) has been retarded after being treated with fenoxycarb (Banu and Manogem, 2022). In essence, the study of ovarian development and the effect of biological factors and abiotic factors has become a core component of research around pest prediction and reproductive regulation.

Despite much research on insect ovarian development, relatively little is known about the reproduction system development of the yellow-spined bamboo locust, *Ceracris kiangsu* Tsai (Orthoptera: Arcypteridae). This locust has historically been regarded as the second worst pest species affecting *Phyllostachys heterocycla* (Carr.) and *Indocalamus tessellatus* (Munro). *C. kiangsu* Tsai has been recorded in East Asian and Southeast Asian countries, such as China, Laos, and Vietnam, with the scales and frequency of *C. kiangsu* population outbreaks increasing gradually, attacking agricultural crops including *Zea mays* L., *Sorghum bicolor* (L.), *Oryza sativa* L., and *Musa basjoo* Siebold (Liu et al., 2021; Li et al., 2022). Since 2014, there have been a large number of *C. kiangsu* outbreaks in Laos and subsequent outbreaks from Vietnam to South China in 2019 (Zhuo et al., 2020). Liu et al. (2021) revealed that during this immigration, *C. kiangsu* had characteristics similar to *Schistocerca gregaria* (Forskål), whose recurrent outbreaks have affected farming systems throughout Northern Africa. Currently, high-resolution fully polarimetric insect radars are being used to accurately assess and forecast the migration patterns of *C. kiangsu*; however, this is a costly process and equipment-intensive management strategy. This study aims to investigate the potential of using ovarian development monitoring in order to predict the reproduction ability and the population dynamics of *C. kiangsu*.

To fully understand the ovarian structure and stages of *C. kiangsu*, adult female insects were dissected during different developmental stages after emergence. These adult female insects were collected from both the laboratory and field populations. The rearing temperatures were kept constant (30°C), while the field population was kept at a variable temperature. The laboratory-rearing population may provide relative complete series development information of adult female insects. Meanwhile, adult female insects from the field population may ensure the enough dissection replication as the beneficial complement. Hence, there may be differences in ovarian development between the two populations. Moreover, if we just dissected the field population in this study, the whole ovarian developmental process may be missed. To rapidly get the immigration information of *S. frugiperda*, the ovarian length and width were used to predict the ovarian developmental stages (Zhao et al., 2019). Therefore, it is vital to infer the relationship between the phenotypic traits and the stages of oviposition and pre-oviposition. Thus, the information will contribute to easily mastering phenotypic fitness estimating the ovarian development to further provide the migratory and prediction information of *C. kiangsu*.

## 2 Materials and methods

### 2.1 Insect source

*C. kiangsu* adult female insects were collected from both the laboratory population and natural bamboo forests. The laboratory population was reared in the Institute of Plant Protection, Chinese Academy of Agricultural Sciences (IPPCAAS), Beijing, China. The egg pods were collected from a natural bamboo habitat, Anhua County, Hunan Province, China (28°62′N; 111°35′E), and then placed in vermiculite. Using the method of Fang et al. (2022), the egg pods were incubated in a chamber (MGC-1000HP-2, Shanghai Yiheng Scientific Instruments Co., Ltd., Shanghai, China) at 30°C ± 1°C and 65 ± 5% RH under a 16:8 h L:D photoperiod. Nymphs were transferred gently into nylon mesh cages (35 cm × 35 cm × 35 cm) with a maximum of approximately 300 nymphs each. Then, one female and two male adults were transferred from the aforementioned nylon mesh cages to a plastic cylinder (φ = 9 cm; height = 17.8 cm) covered with a nylon mesh for air ventilation. The nymphs and adults were reared under the same condition as the eggs’ hatch and were fed with enough fresh wheat and rice leaves.

The field population of *C. kiangsu* was collected from a bamboo forest in Cha’anpu Town, Taoyuan County, Hunan Province, China (28°42′N; 111°12′E), in late July and early August 2022.

### 2.2 Measurements of phenotypic traits and classification of ovaries

For the laboratory population, after adult emergence, approximately 1–3 healthy female adults were measured and dissected every 3 days until all female adults were dead (the 81st day after adult emergence). However, for the field population, approximately 10–30 healthy female adults were measured and dissected every day from 27 July 2022 to 6 August 2022. The peak data on adult emergence were recorded in advance. In total, 51 and 270 female adults were dissected from the laboratory and field populations, respectively.

The dissection process began with the measurement of the body length and body weight of the female adults by using electronic Vernier calipers (0–150 mm, Shanghai Shenhan Measuring Tools Co., Ltd., Shanghai, China) and the electronic balance (PL203, Mettler-Toledo Instruments Shanghai Co., Ltd., Shanghai, China).

Then, the adult female insects were dissected according to Han et al. (2018). The wings and legs were removed, and the dead locust was placed on a dissecting tray with wax and fixed by means of needles through the thorax. An incision was then made on the middle of sternites using surgical scissors. Approximately 6–8 insect needles were placed through the exoskeleton on the left and right sides of the body and into the wax. Care was taken not to damage the internal organs. Finally, the target organ, ovary, was identified under a stereomicroscope (SZ2-ILST, Olympus Co., Tokyo, Japan) and carefully placed in a Petri dish with saline.

The ovaries were photographed using a super-depth-of-field three-dimensional microscopy system (VHX-2000), and the length, width, and cross-sectional area of the ovaries were also measured. The fundamental ovarian structure was similar to that shown in Liu and Lu (1959), and the ovarian development classification methods were according to Han et al. (2018).

### 2.3 Statistical analysis

The phenotypic traits and ovarian developmental parameters were evaluated using a one-way ANOVA (Duncan’s test; *p* < 0.05; SPSS 22.0).

Then, detrended correspondence analysis (DCA; Hill and Gauch, 1980) was used to select the proper constrained ordination method [e.g., redundancy analysis (RDA) and canonical correspondence analysis (CCA)]. Once the axil length of the dataset after DCA is less than 2, RDA can be selected for further analyses according to Nasser et al. (2019). RDA and Pearson’s correlation analysis were used to assess and quantify the relationship between the development duration, days after emergence, body length, body weight of female adults, and ovarian development for the optimization of variables. The variance inflation factor (VIF) value indicates the collinearity among every two independent variables. Four independent variables in this study were less than 10, and independent variables with *p* < 0.05 were regarded as significantly contributed to the variance in the process of ovarian development (Grech et al., 2019).

The partial least squares (PLS; Wold, 1975) regression is a powerful method that can be used to extract a few latent variables with information content, which explain as much of the covariance as possible between the dependent variables and independent variables. The optimal number of latent variables (ONLVs) was determined according to the lowest value of the root mean square error of the cross-validation (RMSECV) *via* the leave-one-out cross-validation (LOOCV) method, to avoid overfitting or underfitting. The coefficient of multiple determinations (*R* ^*2*^), the root mean squared error of prediction (RMSEP), and the variance explained (%) and variable importance in the projection (VIP) were used to evaluate PLS models. The higher *R* ^*2*^, variance explained, and VIP means the PLS model fits better; among them, the threshold score of a VIP is 1.0 (Porker et al., 2020; Wise and Dolan, 2020).

RDA (Oksanen et al., 2022), Pearson’s correlation analysis (*R* core team, 2022), and PLS (Liland et al., 2022) analysis were carried out using R 4.1.3.

## 3 Results

### 3.1 Ovarian development and structure of female adults

**FIGURE 1:**
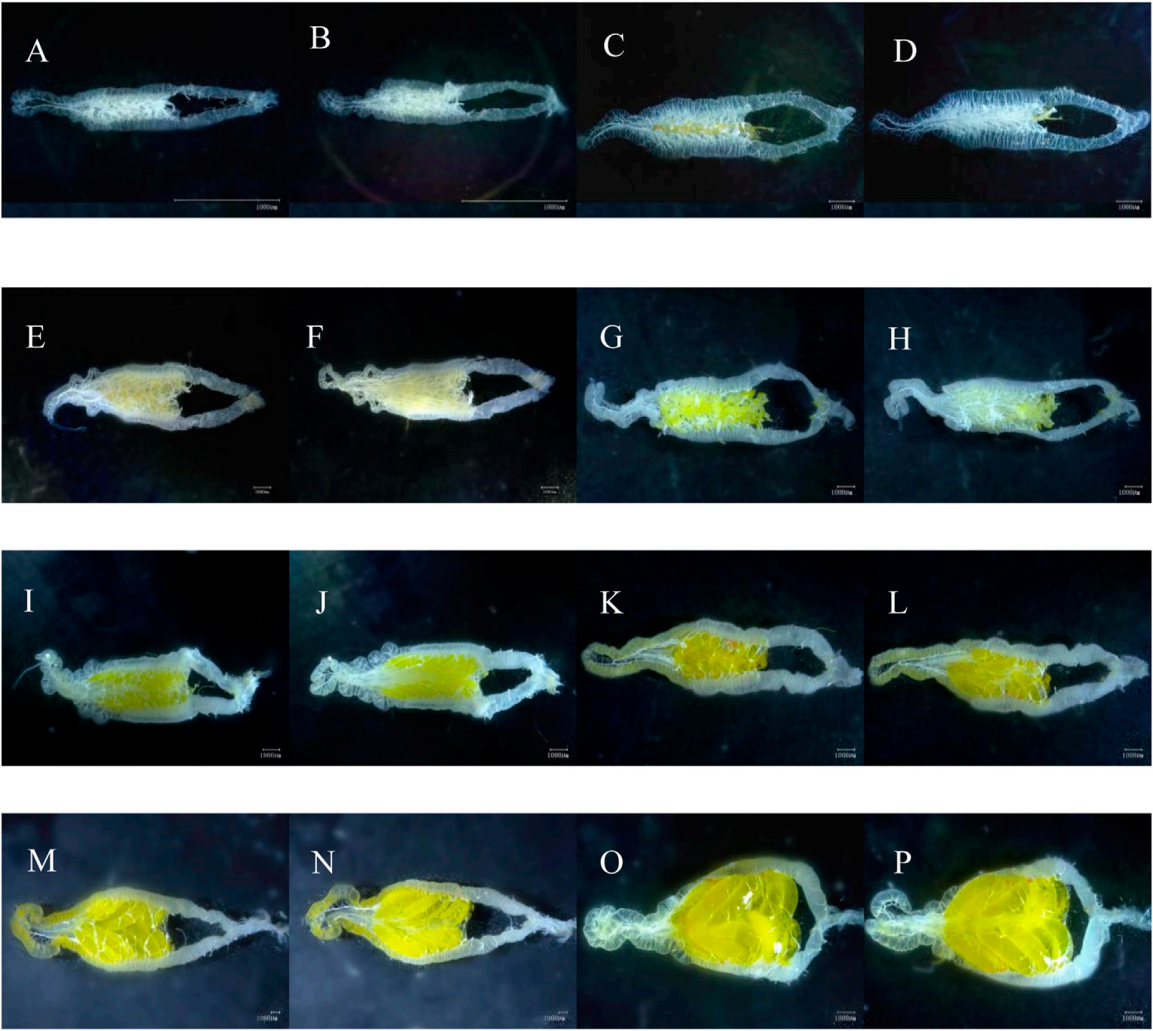
Visual view of *C. kiangsu* ovarian morphology. **(A)** Ventral view at stage Ⅰ, **(B)** dorsal view at stage Ⅰ, **(C)** ventral view at early-stage Ⅱ, **(D)** dorsal view at early-stage Ⅱ, **(E)** ventral view at end-stage Ⅱ, **(F)** dorsal view at end-stage Ⅱ, **(G)** ventral view at early-stage Ⅲ, **(H)** dorsal view at early-stage Ⅲ, **(I)** ventral view at end-stage Ⅲ, **(J)** dorsal view at end-stage Ⅲ, **(K)** ventral view at early-stage Ⅳ, **(L)** dorsal view at early-stage Ⅳ, **(M)** ventral view at end-stage Ⅳ, **(N)** dorsal view at end-stage Ⅳ, **(O)** ventral view at stage Ⅴ, and **(P)** dorsal view at stage Ⅴ (scale bars = 1,000 μm).

In total, 321 adult female insects were dissected over the course of this study. Figure 1 illustrates the development of ovaries with increasing female age. Ovaries shown in Figure 1 were all collected from the laboratory population.

**FIGURE 2:**
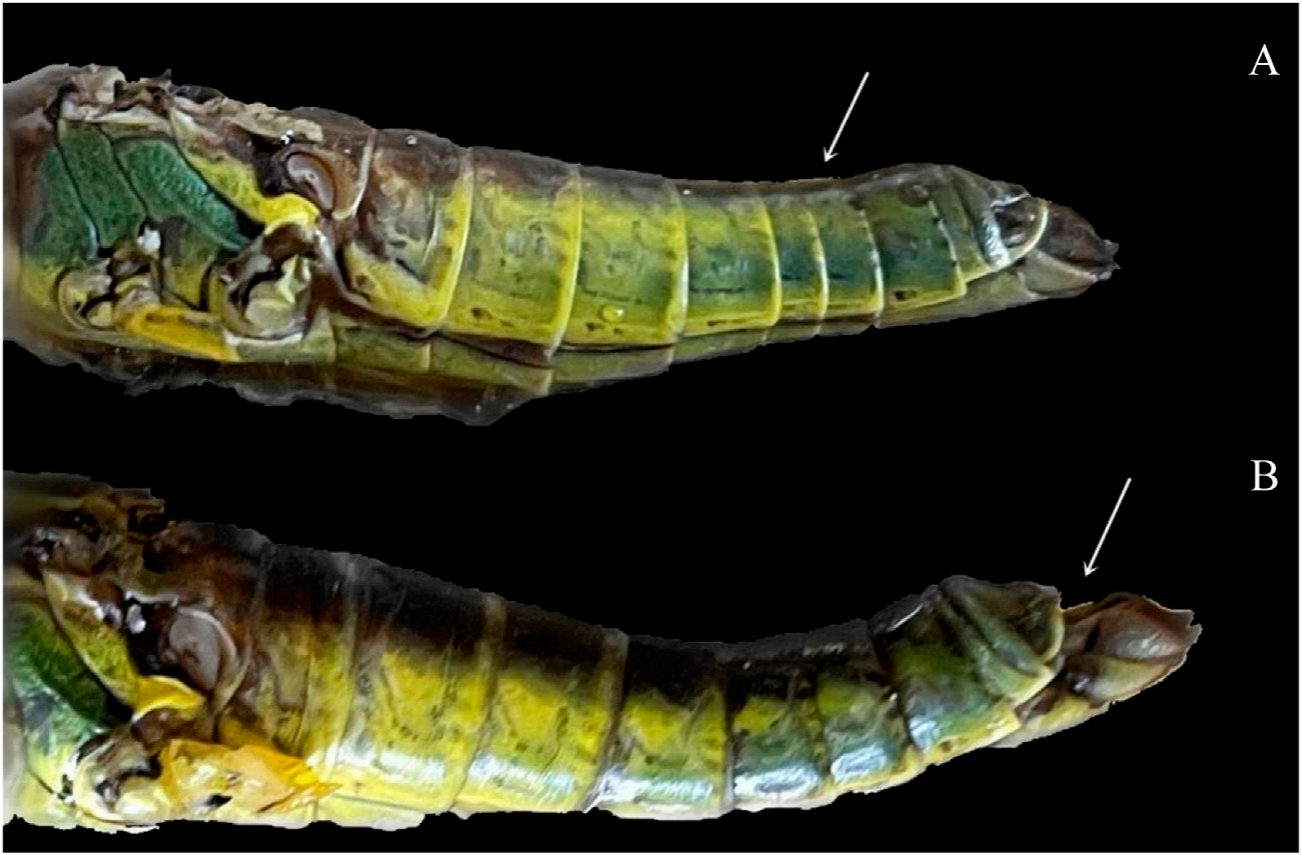
Phenotypic morphology of the *C. kiangsu* abdomen at **(A)** stage Ⅳ and **(B)** stage Ⅴ.

The ovarian development was divided into five stages according to characteristics of ovarioles and oviducts, accessory gland, the number of yolks and eggs, and body fat (Figure 1; Table 1). The main characteristic of ovarian developmental stage I was transparent and thin ovarioles; Figures 1A, B show an ovary from a three-day-old female adult. The main characteristic of ovarian developmental stage II was the appearance of the pale-yellow fat body and yolk, which gradually increased; Figures 1C, D and Figures 1E, F show ovaries from 12-day-old and 18-day-old female adults, respectively. At ovarian developmental stage III, the shape and number of immature eggs appeared and increased in the ovary gradually; Figures 1G, H and Figures 1I, J show ovaries from 27-day-old and 36-day-old female adults, respectively. The main characteristics of stage Ⅳ were the proper arrangement of eggs in the calyx and thickening of the accessory gland. The abdominal morphology showed that the intersegmental membranes between the fifth and sixth segments were folded (Figure 2A); Figures 1K–N show ovaries from 27-day-old and 36-day-old female adults, respectively. The eggs matured, the egg membranes were transparent, and dark red spots appeared in the calyx at stage Ⅴ; Figures 1O, P show the ovary from an 81-day-old female adult. There was an ovipositor located between the eighth and ninth segments. At stage Ⅴ, the valvulae protruded from the end of the abdomen, and the epiproct lifted up (Figure 2B).

**TABLE 1:** Ovarian development characteristics of *C. kiangsu* at different stage standards.

Level	Stage	Characteristics of ovarioles and oviducts	Yolk and egg	Fat body around the ovary	Accessory gland
Ⅰ	Transparency	Ovarioles appeared transparent and slender	NA	NA	Smooth and thin
Ⅱ	Vitellogenesis	Ovarioles appeared white. The lateral oviduct and the median oviduct became thickened and widened	Yolk began to appear and gradually increased	Little pale-yellow fat body	Thickening and folds were deepening
Ⅲ	Egg formation	The calyx formed with the gradually expanding lateral oviducts. The median oviduct became longer	Yolk reduced and eggs appeared	Pale-yellow fat body increased	Folds became deepest
Ⅳ	Pre-oviposition	Ovarioles partially encased the eggs. The median oviduct became longer gradually	Eggs arranged neatly	Orange fat body	Thickening and folds spread
Ⅴ	Oviposition	Ovarioles in the calyx rarely encased eggs	Eggs matured, egg membranes were transparent, and the dark red spots appeared in the calyx	Little orange fat body	The gland appeared the thickest

**FIGURE 3:**
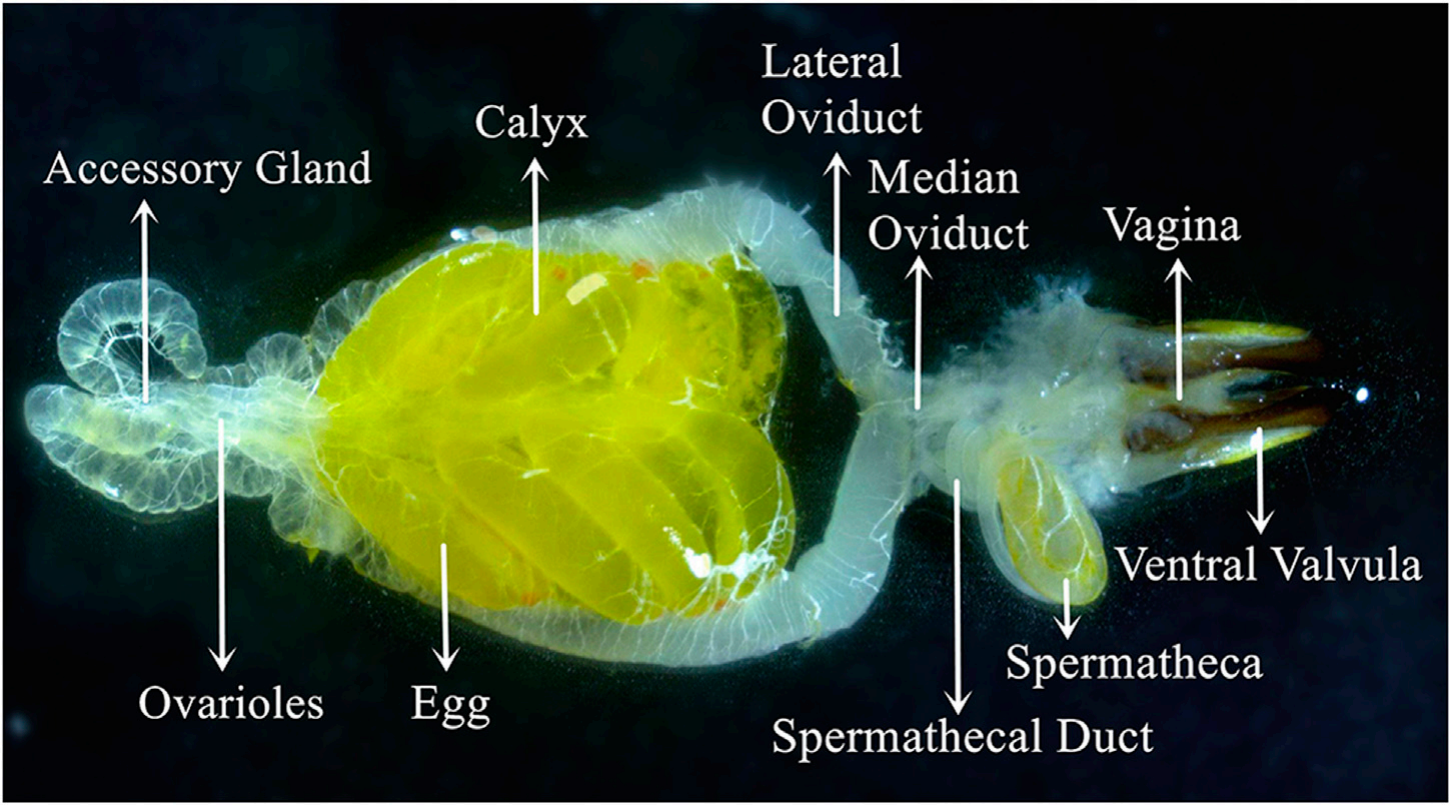
Visual view of the *C. kiangsu* ovary at stage Ⅴ.

The *C. kiangsu* adult female essential reproductive system (Figure 3) consisted of a pair of ovaries. There was a pair of accessory glands connected to a pair of calyxes and lateral oviducts, and the oviducts jointly form the median oviduct, which led to the vagina. The spermatheca connected with the median oviduct and vagina by the spermatheca duct. There was a suspensory ligament located in the anterior end of the accessory gland, and the suspensory ligament was generally thin and transparent and not shown in Figure 3.

### 3.2 Phenotype characteristics of different ovarian developmental stages

**FIGURE 4:**
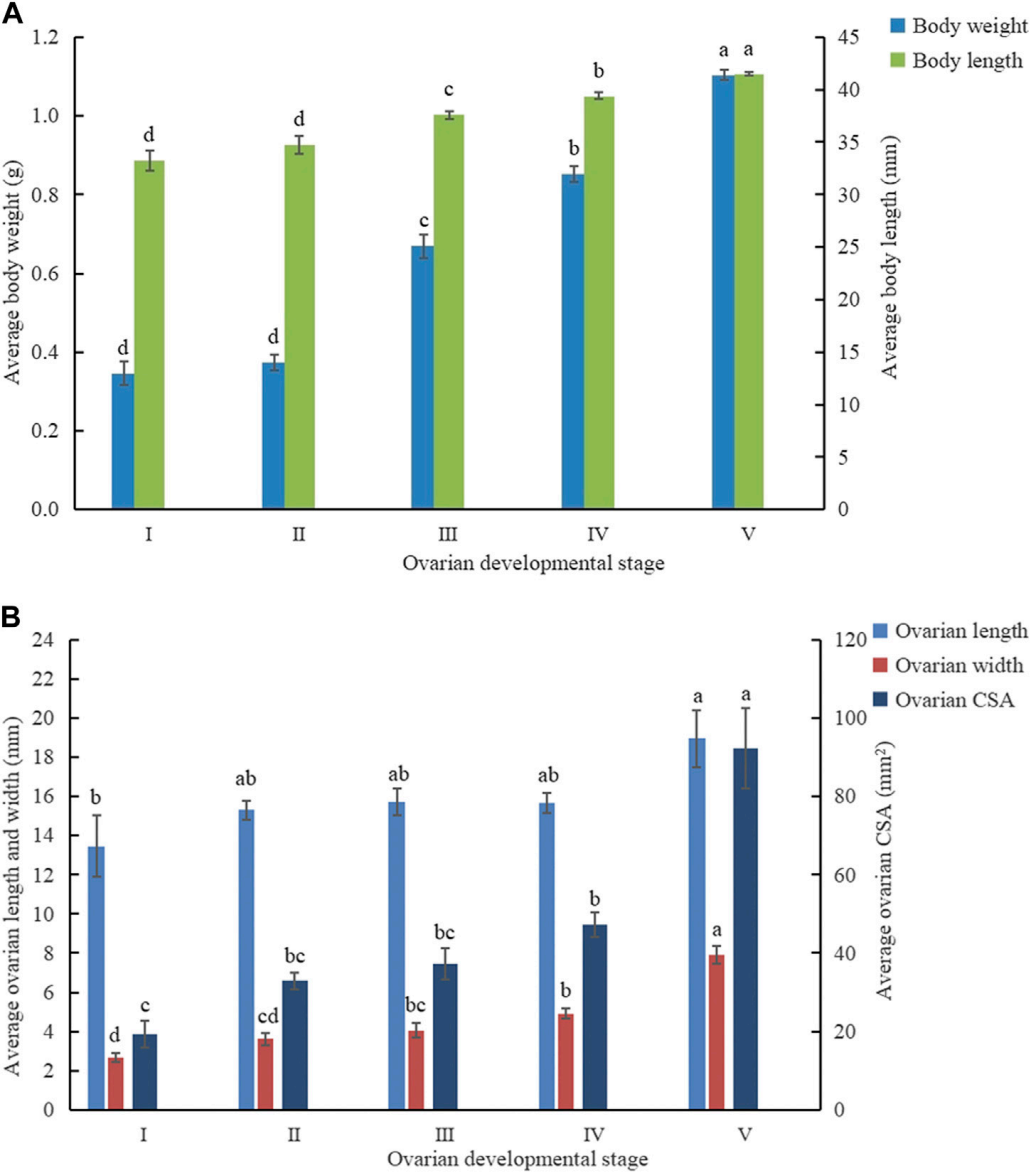
Mean value (±SE) at different ovarian developmental stages of **(A)** phenotypic traits and **(B)** ovarian developmental parameters. CSA, cross-sectional area.

The analysis indicated that there was an increasing tendency of both ovarian and phenotype characteristics with the progression of ovarian development stages as *C. kiangsu* matured (Figure 4). The ovarian width (7.90 ± 0.47 mm) and cross-sectional area (92.25 ± 10.24 mm^2^) at ovarian development stage V were significantly wider and bigger than those at the other four stages, respectively, but there was no significant difference found among the ovarian lengths of ovarian development stages from II to IV. At stage V, the ovarian length was significantly longer than that at stage I (18.95 ± 1.72 mm versus 13.47 ± 1.56 mm).

Similarly, the average body weight and average body length increased proportionally to the ovarian development stage. The average body weight (1.11 ± 0.01 g) and body length (41.49 ± 0.15 mm) at stage V was significantly heavier and longer than those at the other four stages, respectively.

### 3.3 Correlation analysis between ovarian developmental parameters and independent variables

Pearson’s correlation coefficient analysis and RDA were used to investigate the relationship between body weight and body length during the ovarian development of *C. kiangsu*. Four ovarian developmental parameters were used to evaluate the degree of ovarian development in RDA (stage, ovarian length, ovarian width, and ovarian cross-sectional area).

**FIGURE 5:**
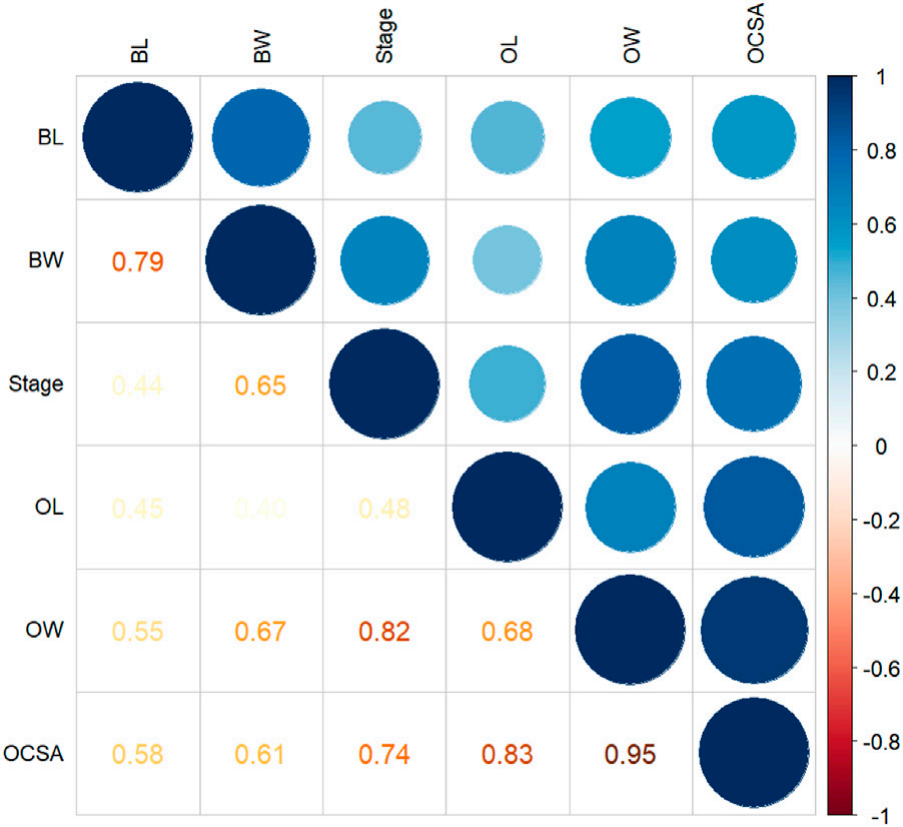
Correlation matrix of all variables. The larger the absolute value and the darker the color, the stronger the Pearson’s correlation (positive or negative). Circles in different colors represent significant correlation; *p <* 0.05. BL, body length; BW, body weight; OL, ovarian length; OW, ovarian width; OCSA, ovarian cross-sectional area.

**FIGURE 6:**
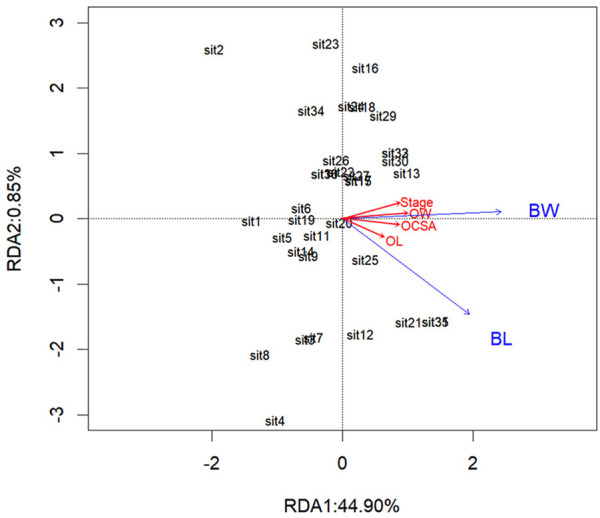
Redundancy analysis diagram of correlations between independent variables and the ovarian developmental parameters. The ovarian developmental parameters are indicated by red color arrows: OL, ovarian length; OW, ovarian width; OCSA, ovarian cross-sectional area. The independent variables are indicated by blue color arrows: BL, body length; BW, body weight.

Pearson’s correlation coefficient matrix (Figure 5) showed that body weight and body length were significantly positively correlated with every ovarian development parameter. Furthermore, RDA (Table 2; Figure 6) showed that both body length and body weight were significantly positively correlated with the degree of ovarian development.

**TABLE 2:** Forward selection results with the test of variable significance.

	RDA 1	RDA 2	Data variance explained (%)	*p*
Body weight/g	0.999	0.040	29.47	0.001***
Body length/mm	0.797	−0.604	12.99	0.009**

*p< 0.05.

RDA showed that body weight and body length accounted for 45.75% of the total variance in the degree of ovarian development, suggesting that the two independent variables had a significant impact on the degree of ovarian development, with Monte Carlo permutation tests with 999 unrestricted permutations (Table 2). The interpretation of the two axes was 44.90% and 0.85%, respectively (Table 3). Therefore, the two axes were selected as the main component axis (Tables 2, 3; Figure 6). From the two independent variables analyzed, the percentage of the total variance explained was the highest for the body weight with 29.47%, which disclosed that body weight had a better explanation of ovarian development than body length (Table 2; Figure 6).

**TABLE 3:** Summary of RDA for trapped samples.

Axis	Eigenvalue	Eigenvalues for the unconstrained axis	Interpretation (%)
Ⅰ	0.050	0.052	44.90
Ⅱ	0.001	0.001	0.85

### 3.4 PLS regression models of ovarian development

**FIGURE 7:**
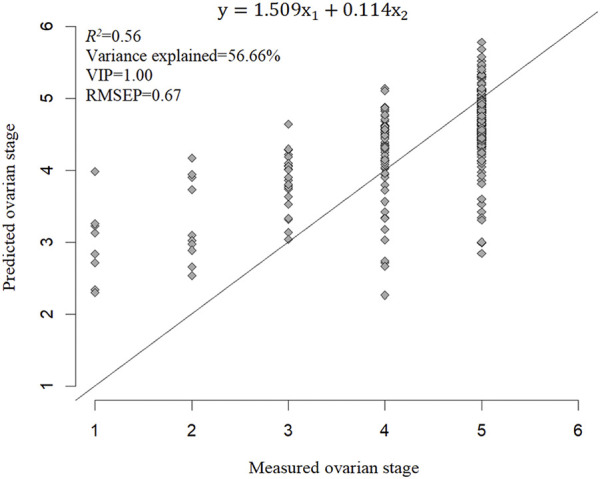
PLS prediction of ovarian developmental parameters for Stage. In this model, y was stage, x_1_, x_2_ were body weight, body length, respectively.

All four ovarian developmental parameters were used to model dependent variables. The ONLV first determined the number of latent variables according to the lowest value of the RMSECV. The first latent variable was selected and then fitted in PLS models again. The optimal models based on each dependent variable and their visualization are shown in Figure 7. The latent variable elucidated 92.41% of the stage-based PLS model. *R* ^*2*^, RMSEP, and VIP of the stage-based PLS model were 0.56, 0.67, and 1.00, respectively, which suggested that the stage-based PLS model fitted better (Figure 7).

Furthermore, the VIP of ovarian length-based, ovarian width-based, and ovarian cross-sectional area-based PLS models were all less than 1. Body weight and body length accounted for 56.66% of the total variance explained. The VIP of the stage-based PLS model was greater than 1, which suggests that this PLS model has a relative application in stages’ prediction in the ovarian development of *C. kiangsu*. The PLS model was subjected to statistical validation tests, and the SE, *t*-value, and *p*-value of the PLS model are shown in Table 4, which revealed that body weight had a significant effect on the ovarian developmental stage, but there was no significant effect found among body lengths of ovarian development stages by modeling.

**TABLE 4:** PLS regression coefficient significance test.

Stage-based PLS model	Regression coefficient		
	SE	*t*	*p*
Body weight	0.079	9.749	***
Body length	0.082	0.153	−

*p< 0.05.

## 4 Discussion

Ovarian development significantly influences the reproduction and expansion capabilities of insect pest populations, and it should be considered when developing sustainable pest management strategies, especially for migratory species (Zhang et al., 2016; Roy et al., 2018). For example, when Masaaki and Seiji (2009) disclosed that the population of *L. m. manilensis* overwintered as adults or eggs by observing the ovarian development on Iheya Island, Japan, it was then this overwinter behavior that provided essential information to better control this population. Similarly, when Elamin et al. (2014) revealed that the ovarian developmental stages and the subsequent fecundity of *Oedaleus senegalensis* (Krauss) depended upon the number and function of the ovarioles, this helped improve the pest management operation in North Kordofan State, Sudan. Whilst this study focused on the ovarian development and grade of *C. kiangsu*, it may also help fill the gap by improving basic ovarian development information and thus the pest management strategies for this species. The relationship between the phenotypic traits and ovarian grade can shed light on the population development of *C. kiangsu*.

Overall, the ovarian structure of *C. kiangsu* can be described as similar to *Omocestus viridulus* (L.), *Euchorthippus unicolor* (Ikonn.), *Euchorthippus vittatus* Zheng, etc. (Zhao and Xi, 2005). With five ovarian developmental stages, *C. kiangsu* was found to be similar to *O. decorus asiaticus* (Bey-Bienko). The ovarian developmental duration of *C. kiangsu* was longer than both gregarious and solitary phases of *O. decorus asiaticus* at stages Ⅲ, Ⅳ, and Ⅴ (Han et al., 2018). The changing trend of the accessory glands was consistent between *C. kiangsu* and *Calliptamus italicus* (L.), whilst the accessory glands folded from stage Ⅱ to stage Ⅳ, and the folds became deepest at stage Ⅲ (Ren et al., 2014). However, there were unique ovarian features specific to *C. kiangsu* that set it apart from other species. The median oviduct of *C. kiangsu* did not fold during ovarian development, which was different from *O. viridulus*, *E. unicolor*, and *E. vittatus* (Zhao and Xi, 2005). There were also dark red dots observed on the matured eggs inside the calyx at stage Ⅴ of a *C. kiangsu* ovary, which is not observed in the other species.

In this study, both the ovarian length and width were positively correlated with the ovarian developmental stages, which is similar to *Tomicus yunnanensis Kirkendall & Faccoli* (Liu et al., 2019). In contrast, during the process of ovarian development, the ovarian length of *Telenomus theophilae* Wu et Chen, *L. m. manilensis*, and *Bactrocera cucurbitae* (Coquillett) increased first and then reduced after they had finished laying eggs (Liu et al., 2006; Ouyang et al., 2014; Kwak et al., 2021). After stage Ⅳ, the body length rapidly increased with the ovarian development for the *C. kiangsu* adult female insects, as the ovipositor reached out from the abdomen. Furthermore, there was a positive correlation between the body weight and ovarian developmental stage, due to both the yolk continually being transformed into eggs and the increased number of eggs during stages Ⅱ–Ⅴ, and this change trend of phenotypic traits on ovarian development was in consistent with *Oxya chinensis* Thunberg and *O. japonica* Thunberg (Wu and Zhang, 1997). The RDA results showed that body weight and body length accounted for 45.75% of the total variance explained in ovarian development, with each factor significantly positively correlating with the ovarian development of *C. kiangsu* (*p* < 0.05). A similar trend has been observed in *Bombus terrestris* L. workers, in which the body weight positively correlated with the ovarian development. (Gosterit et al., 2016).

Generally, during the development of migrating insects, they have been shown to exhibit a delayed ovarian development during migration, which has been classified as reproductive diapause (Qi et al., 2011). However, once they arrived at their destination, the ovaries continued to develop, allowing for successful reproduction (Johnson, 1963). Thus, exploring the ovarian development and mating behaviors of migratory pests has become important in discovering their potential source areas and migratory paths. In order to better implement pest management strategies for *C. kiangsu*, a rapid method to obtain the ovarian developmental stage in the field is fundamental. Previously, PLS was used to predict either continuous or discrete variables (Elsherif et al., 2018); however, it can also be applied to pest management. For example, Xu et al. (2020) used this approach to produce a predictive diagnostic model for cotton aphids based on leaf textural features; the predictive accuracy of up to 91.49% was obtained for this model. In this study, in order to rapidly obtain the degree of ovarian development of *C. kiangsu* without dissection, body weight and body length were used as predictors. The model parameter standards showed that stage-based PLS modeling was the preferred approach, which can be used in a predictive capacity, and thus, it could potentially be a reference model to identify whether the field populations are “native” or “migratory.”

The ovarian development process is highly dynamic (Papaj, 2000). Berger et al. (2008) found that a heavier female insect usually had a higher potential fecundity, and different sizes of female insects indicated different allocation trade-offs between the self-condition and their egg production. However, this phenomenon is found in reproductive systems of not only female insects but also male insects (Leopold, 1976). Chen et al. (2019) showed that the mating rate in the current generation can be reduced through sex pheromone-trapped male adults, which is then reflected by the size of the *Spodoptera litura* male testis. Hence, the observation of the reproductive system of migratory insects is a determining factor in determining the origin and degree of development of migratory insects. The studies can provide information on integrated pest management (IPM) of migrants, but these aforementioned studies of *C. kiangsu* were much less explored.

This study identified the ovarian development process of *C. kiangsu* adult female insects. By grading the ovarian developmental stages, it is easier to identify the transition and critical time between different stages, thus improving the prediction accuracy of the population development of *C. kiangsu*. Moreover, it could be helpful in the study of migratory pathways and predicting the occurrence of pests. It also provides additional information about the further exploration of *C. kiangsu* reproductive systems.

## Data Availability

The raw data supporting the conclusion of this article will be made available by the authors, without undue reservation.
